# Promoting the expansion and function of human corneal endothelial cells with an orbital adipose-derived stem cell-conditioned medium

**DOI:** 10.1186/s13287-017-0737-5

**Published:** 2017-12-20

**Authors:** Peng Sun, Lin Shen, Canwei Zhang, Liqun Du, Xinyi Wu

**Affiliations:** 1 Department of Ophthalmology, Qilu Hospital of Shandong University, Jinan, Shandong 250012 People’s Republic of China; 2 The Key Laboratory of Cardiovascular Remodeling and Function Research, Chinese Ministry of Education and Chinese Ministry of Health, Qilu Hospital of Shandong University, Jinan, Shandong 250012 People’s Republic of China

**Keywords:** Corneal endothelial cell, Orbital adipose-derived stem cell, Conditioned medium, Corneal endothelial dysfunction, Regenerative medicine, Tissue engineering, Animal model, Cell therapy

## Abstract

**Background:**

Corneal endothelial dysfunction causes severe impairment of vision. The only solution is corneal transplantation. However, this treatment is hampered by a worldwide shortage of donor corneas. New therapies may replace the conventional donor corneal transplantation alongside the developments in regenerative medicine and tissue engineering, but sufficient functional corneal endothelial cells (CECs) are essential. The aim of this study was to promote the expansion and function of human corneal endothelial cells (HCECs) in vitro and in vivo.

**Methods:**

The phenotypes of human orbital adipose-derived stem cells (OASCs) were detected by flow cytometry and immunofluorescence. HCECs were isolated and cultured using a conditioned medium obtained from OASCs (OASC-CM) in vitro. Related cell markers of HCECs were analyzed by quantitative real-time polymerase chain reaction (qRT-PCR), Western blot, and immunofluorescence. The cell counting kit-8 (CCK-8) assay and the wound healing assay were performed to evaluate the proliferation ability of the cells. The cultured HCECs were then transplanted into rabbit and monkey corneal endothelial dysfunction models by cell injection.

**Results:**

CD29, CD105, CD49e, CD166, and vimentin were highly expressed in cultured human OASCs. The CEC-relative markers zonula occludens-1 (ZO-1), Na^+^/K^+^ ATPase, N-cadherin, Col8a2, and SLC4A4 were expressed in HCECs cultured by OASC-CM. The HCECs were able to maintain polygonal cell morphology and good proliferative capacity. In animal experiments, corneal transparency was achieved after the injection of HCECs, which demonstrated the good repair capacity of the cells.

**Conclusions:**

The proliferation abilities of the cells were significantly enhanced, and related functional markers were strongly positive, while HCEC morphology was maintained using OASC-CM. HCECs obtained some stem cell-like properties. This preclinical study confirmed the therapeutic ability of the HCECs in vivo. Our findings demonstrated that cultured HCECs with OASC-CM might be a promising source for research and clinical treatment.

**Electronic supplementary material:**

The online version of this article (doi:10.1186/s13287-017-0737-5) contains supplementary material, which is available to authorized users.

## Background

The corneal endothelium (CE) plays a very important role in maintaining cornea transparency via pump and barrier functions [1]. Corneal endothelial cells (CECs) have limited proliferative capacity in vivo [2–4]. Consequently, endothelial dysfunction causing severe vision loss is unavoidable when the number of endothelial cells reaches a critical cell density (<1000 cells/mm^2^) due to a pathological status such as Fuchs endothelial corneal dystrophies, intraocular surgery, or trauma [1, 5]. Corneal transplantation (full corneal transplant or endothelial keratoplasty) is currently the only procedure for treating corneal endothelial dysfunction [6, 7]. The treatment relies on a sufficient number of functional human corneal endothelial cells (HCECs). However, the worldwide shortage of transplantable donor corneal tissues remains a big problem [8]. Therefore, researchers are making great efforts using regenerative medicine and tissue engineering to overcome this problem [9]. Researchers have induced CEC-like cells from other types of cells and some of these cells have functions similar to CECs [10–12]. However, the proliferation ability and function of these cells is very limited. Thus there is a pressing need to find optimum protocols to expand HCECs in vitro since the procedures involved in the isolation and subsequent cultivation protocols greatly vary between laboratories [13–16].

In the present study, we first used conditioned medium (CM) obtained from human orbital adipose-derived stem cells (OASCs; OASC-CM) when cultivating HCECs. The cells were then investigated by proliferation assay in a preclinical study. Our findings showed that HCECs could maintain quite a good proliferative and cell-based therapeutic capacity even after 10 passages. These studies indicated that more useful cells should be available for the research into HCECs and clinical cell-based therapy for corneal endothelial dysfunction in the future.

## Methods

### Animals

Thirty-five New Zealand white rabbits weighing 2.0–4.0 kg (Xilingjiao Experimental Animal Breeding Center, Jinan, Shandong Province, China) and six rhesus monkeys weighing 3.0–4.0 kg (3 to 5 years of age; Hongli Medical Animal Experimental Research Center, Jinan, Shandong Province, China) were used for animal experiments.

### Isolation and primary culture of OASCs and HCECs

Redundant orbital adipose tissues were collected from 15 patients aged between 23 and 65 (45.3 ± 9.8) years following blepharoplastic surgeries. The OASCs were cultivated as previously described with slight modification [17, 18]. Briefly, the orbital adipose tissues were repeatedly washed with phosphate-buffered saline (PBS). The tissues were then fragmented with surgical scissors and suspended in 0.1% collagenase type I (Sigma) at 37 °C with gentle stirring. After 2–4 h digestion, fragmented tissues were re-suspended in Dulbecco’s modified Eagle’s medium low glucose (DMEM-LG) containing 10% fetal bovine serum (FBS) for 5 min at room temperature (RT) and then filtered through a 70-μm strainer. The fluid was washed with PBS and centrifuged twice for 5 min at 1200 rpm at RT. Cell suspensions were plated in T-25 flasks (Corning) in DMEM-LG supplemented with 10% FBS (Gibco) and 10% penicillin-streptomycin (Sigma), and incubated at 37 °C in 5% CO_2_. After 2–4 days, nonadherent cells were removed, and adherent cells continued to be cultured until they reached confluence. The OASCs at passages 3 through 8 were used for experiments.

HCECs were obtained from discarded corneal-scleral rings after penetrating keratoplasty (PK) and from the Eye Tissue Bank of Shandong Province, China. The age of donors ranged from 15 to 78 years (*n* = 10). Cells were cultured in accordance with previously published methods with some modification [13]. Briefly, after corneas were washed three times with M199 (Hyclone), the Descemet’s membranes (DM) containing HCECs were stripped and incubated in basal culture medium (BM) overnight at 37 °C in 5% CO_2_ for stabilization, followed by digestion with 1 mg/mL collagenase type I (Sigma) for 1–2 h. The HCECs were re-suspended and seeded in one well of a 12-well plate coated with FNC Coating Mix (Usbio). The cells were cultured in BM (BM-HCECs) as the control group and in BM containing 10% OASC-CM (CM-HCECs) as the experimental group. The BM was composed of Opti-MEM-I (Gibco), 8% FBS, 5 ng/mL human epidermal growth factor (hEGF; PeproTech), 20 μg/mL ascorbic acid (Sigma), 200 mg/L calcium chloride, 0.08% chondroitin sulfate, and 50 μg/mL penicillin-streptomycin [19].

### Flow cytometry

Related cell markers of OASCs were analyzed by flow cytometry. The dissociated cells were incubated with fluorescein isothiocyanate (FITC) mouse anti-human CD29, phycoerythrin (PE) mouse anti-human CD34, PE mouse anti-human CD18, FITC mouse anti-human CD49e, PE mouse anti-human CD166, allophycocyanin (APC) mouse anti-human CD133, PE mouse anti-human CD45, and APC mouse anti-human CD105 (BD Biosciences) respectively at 4 °C for 30 min, washed, and resuspended with PBS. The cells then underwent flow cytometry using the BD FACS Calibur. Analysis was performed using the Flow-Jo program (Treestar, USA).

### Preparation of OASC-CM

OASCs were washed three times with PBS when they were at 60–80% confluence and the medium was replaced with basal growth medium. The OASCs were kept for an additional 12–24 h. The medium was then collected and filtered (0.22 μm) and stored at −80 °C to preserve the biological activity.

### Cell proliferation with a cell counting kit-8 (CCK-8) assay

Cell viability was measured using a commercial CCK-8 assay kit (BestBio Science, Shanghai, China) according to the manufacturer’s protocol. Cells (7 × 10^3^ cells per well) were placed in 96-well plates with BM overnight at 37 °C in 5% CO_2_. The BM was then removed. Cells were cultured in medium with different concentrations of OASC-CM for 24 h at 37 °C in 5% CO_2_. CCK-8 solution (10 μl) was added to each well, and cells were incubated at 37 °C for 2.5 h. The absorbance at 450 nm was measured using a multimode reader (MD).

### Immunofluorescence

Cell or tissue sections were fixed with 4% paraformaldehyde for 15 min and incubated in 0.5% Triton X-100 for 10 min and 5% goat serum for 30 min. Primary antibodies were mouse anti-N-cadherin (1:100; Abcam), mouse anti-Na^+^/K^+^ ATPase α-1 (1:200; Millipore), rabbit anti-zonula occludens-1 (ZO-1; 1:100; Santa Cruz), mouse anti-human nuclei (1:100; Millipore), and rabbit anti-vimentin (1:200; Abcam). Cells were incubated with the primary antibodies overnight at 4 °C. Secondary antibodies (1:100; all obtained from Beijing Zhongshan Technologies) coupled to FITC or TRITC were then applied for detection. Subsequently, the cells were stained with DAPI to visualize the nuclei. In negative controls, primary antibodies were substituted by PBS. Fluorescence was observed using a fluorescent microscope.

### Western blotting

The total protein of HCECs was extracted using 1% radioimmunoprecipitation assay lysis buffer (Beyotime) and quantified with the bicinchoninic acid protein assay kit (Beyotime). The protein was loaded onto a 10% sodium dodecyl sulfate-polyacrylamide electrophoresis gel, electrophoresed, and then transferred to a polyvinylidene difluoride membrane (Millipore). Membranes were blocked with 5% nonfat dried milk at room temperature for 2 h, washed three times with TBST, and incubated with the primary antibody against rabbit anti-Na^+^/K^+^ ATPase α1 (1:100; CST), rabbit anti-ZO-1 (1:1000, CST), and GAPDH (1:1000; Beijing Zhongshan) overnight at 4 °C. After immunoblotting with secondary antibodies (1:5000; Beyotime) at room temperature for 1 h, the protein was detected with an enhanced chemiluminescent reagent (Millipore).

### Quantitative real-time reverse transcription polymerase chain (qRT-PCR) reaction

The total RNA was extracted from HCECs using Trizol Reagent (Invitrogen) according to the manufacturer’s protocol. A 1-μg sample of total RNA was reverse-transcribed to cDNA (complimentary DNA) with the ReverTra Ace kit (Toyobo). PCR analysis was performed using the SYBR Green enzyme mixture (Toyobo) and the Applied Roche 480 Real-Time PCR system according to the manufacturer’s protocol. Primers used in the PCR are described in Table 1.

**Table 1:** List of primers for polymerase chain reaction

Gene	Primer	Sequence (5′–3′)	Size (bp)
Na^+^/K^+^ ATPase α 1	Forward	CTGTGGATTGGAGCGATTCTT	112
	Reverse	TTACAACGGCTGATAGCACCA	
Zo-1	Forward	ATCCCTATCACCCAGCGTCA	152
	Reverse	TCTCCCACTCTGTCTCCAGG	
Col8a2	Forward	CGACCTGAAAGCACGTCCAC	85
	Reverse	AGAGGCATTTCAGTAGCAGCA	
SLC4A4	Forward	TGATCGGGAGGCTTCTTCTCT	154
	Reverse	GGACCGAAGGTTGGATTTCTTG	
GAPDH	Forward	GCACCGTCAAGGCTGAGAAC	138
	Reverse	TGGTGAAGACGCCAGTGGA	

### Wound healing assay

A wound healing assay was used to the compare cell migration and repair capacity of HCECs of different passages. HCECs were cultured in six-well plates in BM or CM. Linear wounds were created by scraping confluent cell monolayers with the tips of sterile pipettes. The detached cells were rinsed away with PBS after the scratch and the remaining HCECs were cultivated with medium. Phase-contrast pictures of the wound recovery were taken at 0, 3, 6, 9, and 12 h. The remaining wound area was measured using Image J software.

### Injection of HCECs into a rabbit corneal endothelial dysfunction model

Cell injection was performed to evaluate the cell-based therapy of HCECs. The cells were labeled by carboxyfluorescein diacetate succinimdyl ester (CFSE; Gene Copoeia) according to the manufacturer’s protocol. Thirty-five rabbits were randomly divided into three groups: the CM-HCEC group, the BM-HCEC group, and the control group. The rabbits were intravenously anesthetized with 3% pentobarbital sodium and topically with oxybuprocaine hydrochloride. The corneal endothelial dysfunction model was created according to a previous method with slight modification [20, 21] (Additional file 1: Figure S1A and C). A modified irrigator needle (Shandong Weigao) was used to completely scrape the CE from the DM (Additional file 1: Figure S1B). The aqueous humor was collected after anterior chamber irrigation; part of this was stained by trypan blue and hematoxylin to detect residual cells. The other part was placed in 96-well plates with culture medium at 37 °C in 5% CO_2_ and observed daily by microscope.

Before cell injection, 100 μl aqueous humor was extracted from the anterior chamber of the rabbit corneal endothelial dysfunction models. CFSE-labeled HCECs (3.0 × 10^5^ cells) were then suspended in 100 μl Opti-MEM-I and injected into the anterior chamber of the eyes using an insulin needle (BD). A peribulbar injection of triamcinolone acetonide and a subconjuctival injection of dexamethasone were given. After the injection, the operation eyes were kept in the face-down position for 5 h under general anesthesia. Tobramycin and dexamethasone drops were also given topically four times a day. Cultivated HCECs with BM at passage 3 (P3) were injected into the right eyes of five rabbits as the BM-HCEC group. Cultivated HCECs with CM from passage 7 to passage 13 (P7, P9, P11, and P13) were injected into the right eyes of another 20 rabbits, respectively. These 20 rabbits were used as the CM-HCEC group. The left eyes of the rabbits were observed as the normal control. The left eyes of the remaining 10 rabbits in which the CE was mechanically removed without cell injection were used as the control group.

The corneal transparency and thickness were observed and photographed using slit-lamp microscopy (Topcon) and optical coherent tomography (OCT; Carl Zeiss). Images of CECs were taken and analyzed by confocal microscopy (HRT-II, Heidelberg Engineering) at days 1, 3, and 7.

### Histological examination of rabbit eyes

Rabbits were sacrificed by an intravenous overdose of pentobarbital sodium 3 and 7 days after the transplantation. Postoperative eyes were removed and part of the cornea was embedded in Tissue-Tek optimum cutting temperature compound (Sakura) and sectioned into 5-μm slices. The frozen slices were viewed under a microscope to detect the CFSE signal and were subjected to immunofluorescent staining. Part of the cornea was stained with Alizarin Red S and trypan blue to show cell survival and borders. Another part of the cornea was fixed in 4% formaldehyde and subjected to hematoxylin and eosin (H&E) staining.

### Injection of HCECs into a monkey corneal endothelial dysfunction model

Six monkeys were randomly divided into the CM-HCEC group (*n* = 4) and the control group (*n* = 2). Monkeys were placed under general anesthesia using ketamine hydrochloride. Monkey corneal endothelial pathological dysfunction models were created in the same manner as the rabbit models (Additional file 1: Figure S1A and D). In the preliminary experiments on the rabbits, we found that the corneal opacity could not be completely cured by BM-HCECs and CM-HCECs after P13, so we used P9 and P11 of CM-HCECs for cell injection.

Aqueous humor (50 μl) was first extracted from the anterior chamber. CFSE-labeled HCECs (2.7 × 10^5^ cells for each eye) were suspended in 50 μl Opti-MEM-I and injected into the anterior chamber of four monkeys as the CM-HCEC group. The other two monkeys had the CE removed as the control group. The nonsurgical eyes of all monkeys were used as the normal group. A peribulbar injection of triamcinolone acetonide and a subconjuctival injection of dexamethasone were given. After the injection, the operation eyes were kept in a face-down position for 5 h under general anesthesia. Tobramycin and dexamethasone drops were given four times a day. The corneas were examined by a slit-lamp microscopy, AccuPen Handheld Tonometer (Accutome), OCT, confocal microscope, and noncontact specular microscopy (Topcon) at certain times after surgery. One monkey from the CM-HCEC group was euthanized at 2 months after the injection. Postoperative eyes were removed and part of the cornea was used to detect the CFSE signal and had immunofluorescent staining in the frozen section, and the other part was subjected to H&E staining.

### Statistical analysis

Data are presented as the means ± SEM. The Student’s *t* test was used to examine differences between the two groups. Comparisons among three or more groups were made using one-way analysis of variance (ANOVA) and post-hoc analysis with the Bonferroni test. Differences were considered to be statistically significant at *p* < 0.05.

## Results

### Characterization of human OASCs

**Fig. 1:**
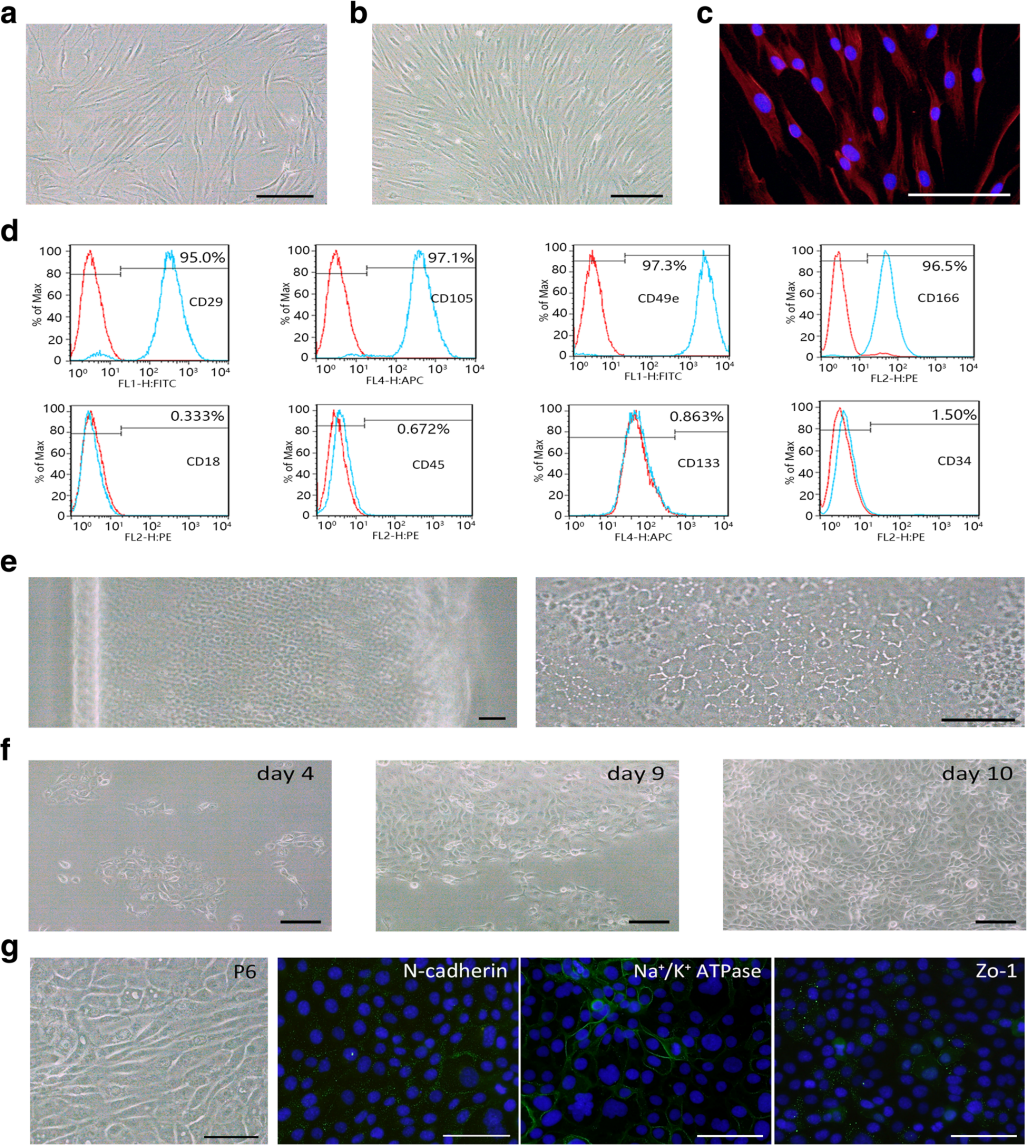
Isolation, culture, and characteristics of human orbital adipose-derived stem cells (OASCs) and human corneal endothelial cells (HCECs). **a**,**b** OASCs were adherent, spindle-shaped, fibroblast-like cells. **c** Immunofluorescence staining of vimentin. **d** Flow cytometric analyses of cell markers in human OASCs (*n* = 3). *Blue lines* indicate the OASC group. **e** Peeled DM layer that contained endothelial cells (*left panel*) and a magnification micrograph of the DM layer (*right panel*). **f** Primary culture of HCECs with OASC-CM (days 4, 9, and 10). **g** Fibroblastic conversion and expression of CEC relative markers in P6 BM-HCECs. *Scale bars* = 100 μm

In our study, human OASCs were plastic adherent, spindle-shaped, fibroblast-like cells (Fig. 1a and b). Surface immunophenotyping characterized by flow cytometry revealed that OASCs were negative for the bone marrow stromal cell marker CD18, the leukocyte common antigen CD45, the hematopoietic stem cell marker CD34, and CD133, and highly expressed CD29, CD105, CD49e, and CD166 (Fig. 1d), suggesting their endothelial and stem cell origin. Immunofluorescence staining showed that OASCs strongly expressed vimentin in the cell plasma which is a kind of intermediate filament protein found in normal HCECs (Fig. 1c) [22]. Cultured OASCs demonstrated similar morphology and phenotype before passage 10.

### Effect of OASC-CM on the proliferation and expansion of HCECs in vitro

**Fig. 2:**
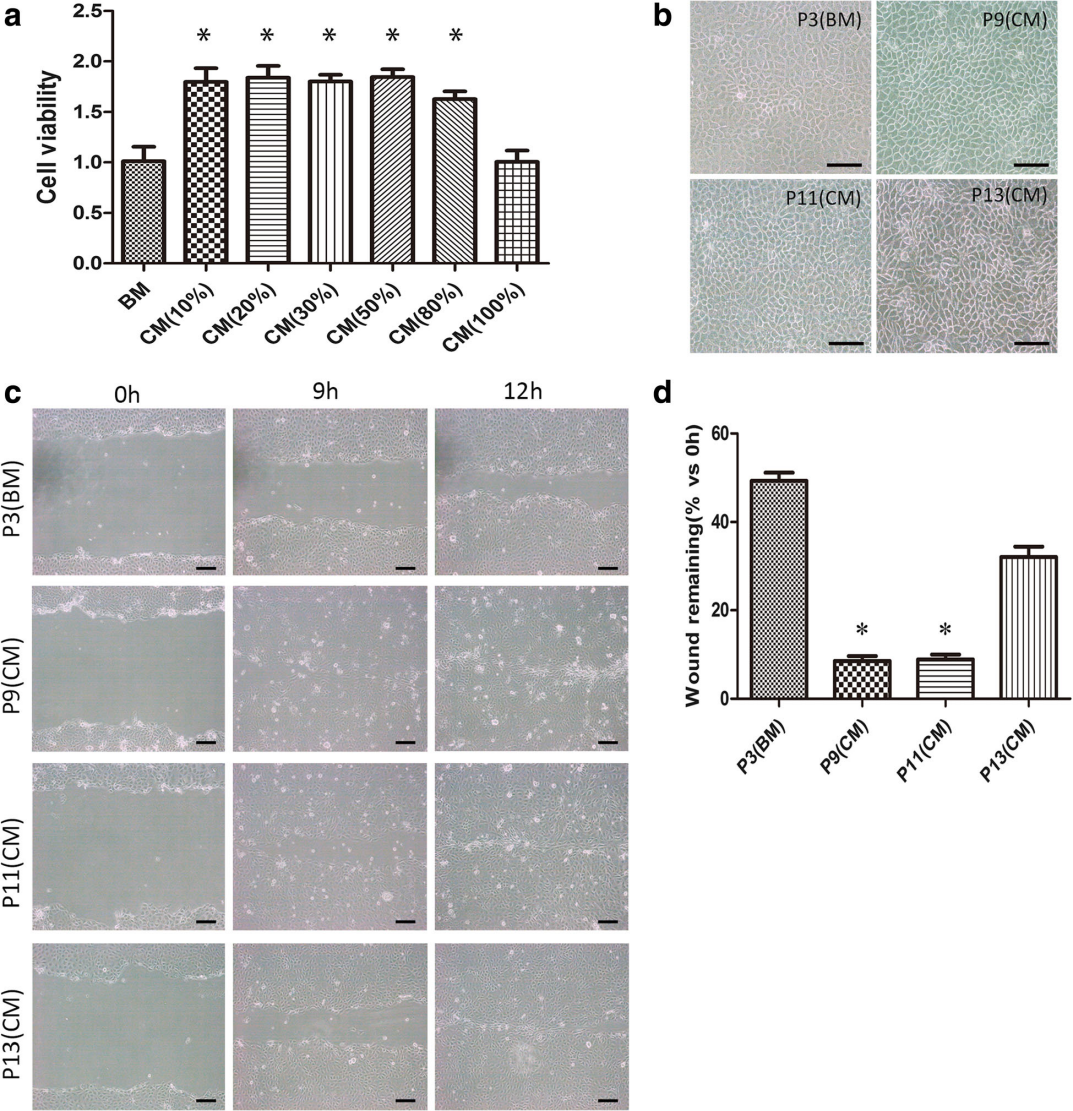
OASC-CM enhances the proliferation of HCECs. **a** The effect of OASC-CM on the proliferation of HCECs was evaluated by cell counting kit-8 (CCK-8) assay (*n* = 3). **b** The morphology of different passages of HCECs cultured in BM or OASC-CM. **c** Proliferative and therapeutic capacity of HCECs were compared by wound healing assay. **d** The remaining wound area was measured by Image J software after 9 h. Results are means ± SEM (*n* = 3). *p < 0.05, versus P3 (BM). *Scale bars* = 100 μm. *BM* basal medium, *CM* conditioned medium, *P* passage

HCECs displayed hexagonal morphology on the DM in vivo (Fig. 1e). Under phase-contrast microscopy, the cultured primary HCECs passage 0 (P0) had a mosaic pattern (Fig. 1f), but after 4–5 passages cultured in BM HCECs began a fibroblastic change, became larger with vacuoles, and showed endothelial-to-mesenchymal transition (EMT). Expression levels of CEC-relative markers were also significantly decreased (Fig. 1g). We used OASC-CM and evaluated the effect on proliferation of HCECs. First, to test the suitable dilution, OASC-CM was added to the BM at final concentrations of 10%, 20% 30%, 50%, 80%, or 100%. The proliferative activity of the OASC-CM was then compared to that of the BM by CCK-8 assay. The assay revealed that the proliferation of HCECs in the BM group was significantly lower than that in the 10–80% strength OASC-CM group. HCECs cultured in 10–80% strength OASC-CM showed no difference in cell proliferation (Fig. 2a), and so we used the concentration of 10% OASC-CM in the following study.

### Effect of OASC-CM on the phenotype of HCECs

**Fig. 3:**
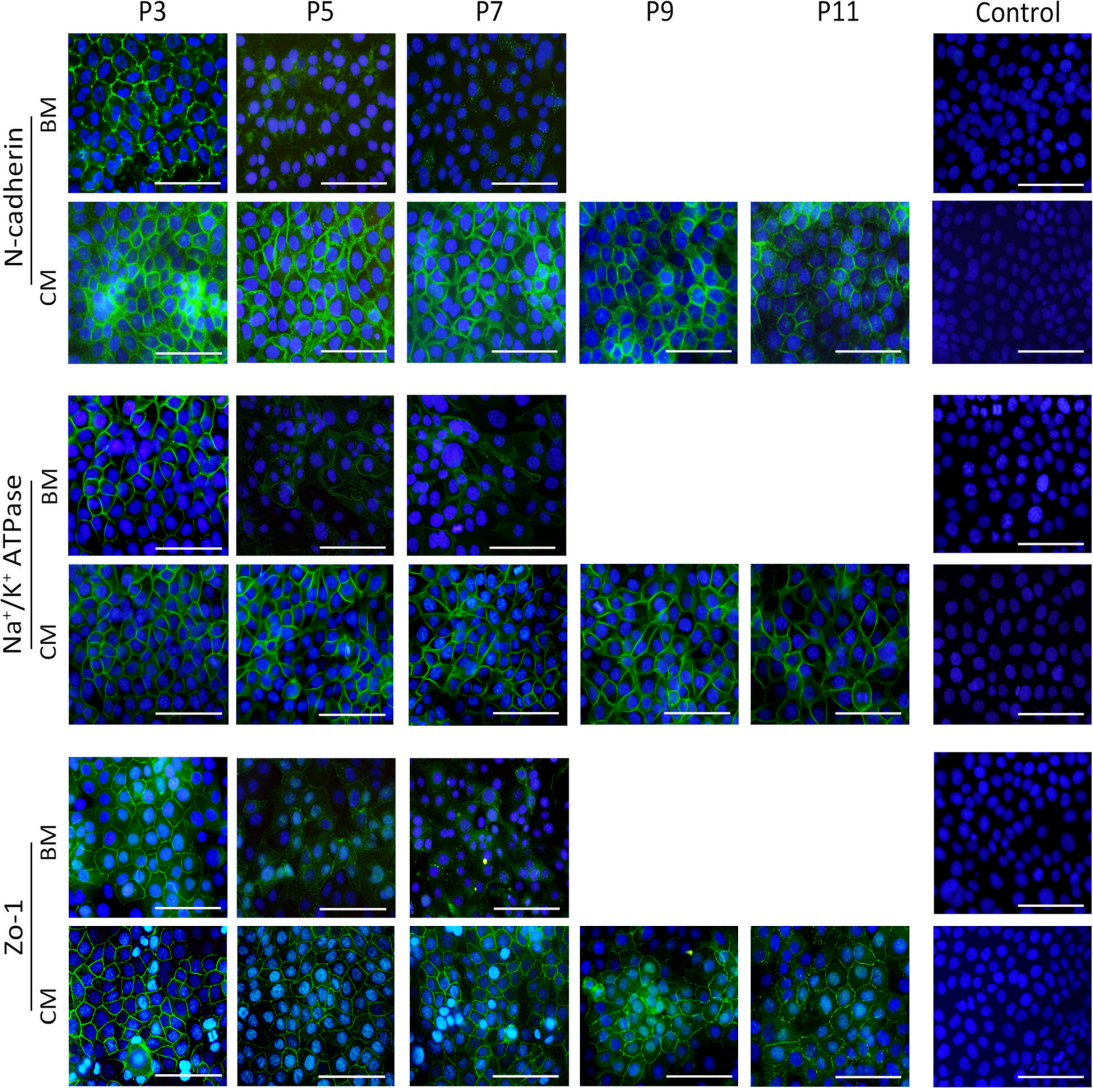
Cell surface marker expression in HCECs at different passages cultivated in BM or OASC-CM. Immunofluorescence staining of N-cadherin, Na^+^/K^+^ ATPase, and ZO-1. *Scale bars* = 100 μm. *BM* basal medium, *CM* conditioned medium, *P* passage, *Zo-1* zonula occludens-1

**Fig. 4:**
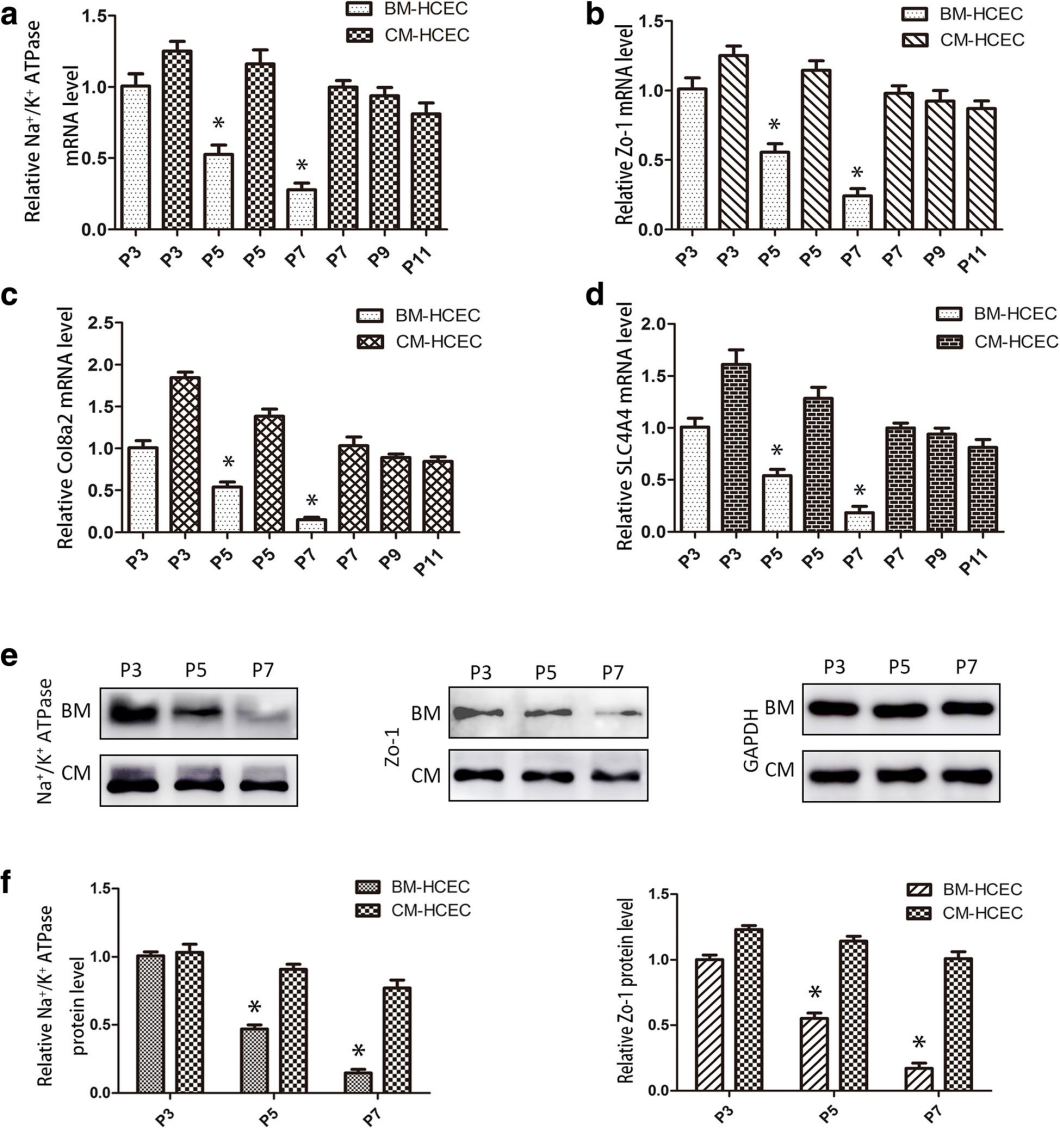
Relative cell marker expression in HCECs at different passages cultivated in BM or OASC-CM. RT-PCR analysis of Na^+^/K^+^ ATPase (**a**), ZO-1 (**b**), Col8a2 (**c**), and SLC4A4 (**d**) (*n* = 3). **e**,**f** Western blotting analysis and protein levels of Na^+^/K^+^ ATPase and ZO-1. Results are means ± SEM (*n* = 3). *p < 0.05, versus P3 (BM). *BM* basal medium, *CM* conditioned medium, *Col8a2* collagen type VIII, *HCEC* human corneal endothelial cell, *P* passage, *SLC4A4* solute carrier family 4 anion exchanger member 4, *Zo-1* zonula occludens-1

The cells cultured in OASC-CM maintained the characteristic polygonal cell morphology and a contact-inhibited monolayer even after 10 passages (Fig. 2b). We then evaluated whether the cells still expressed the CEC-related functional proteins: the corneal endothelial differentiation marker N-cadherin [11, 23], sodium-potassium pump Na^+^/K^+^ ATPase, tight junction protein ZO-1 [10], collagen type VIII (Col8a2), and solute carrier family 4 anion exchanger member 4 (SLC4A4) [24, 25]. Immunostaining of N-cadherin, Na+/K+ ATPase, and ZO-1 clearly outlined the intercellular adherent junction and cell borders of CM-HCECs, while the expressions were very weak in P5 and P7 BM-HCECs (Fig. 3). PCR showed that CM-HCECs expressed high levels of the CEC markers (Fig. 4a–d). Western blot analysis also showed that P3, P5, and P7 CM-HCECs expressed high protein levels of Na^+^/K^+^ ATPase and ZO-1 when similar passages were compared between BM-HCECs and CM-HCECs (Fig. 4e and f).

### Wound healing

We compared four different passages of HCECs (P3 of BM-HCECs as the control and P9, P11, and P13 of CM-HCECs) in the wound healing assay. The wound closure rates were different. CM-HCECs (P9 and P11) demonstrated a faster healing rate (Fig. 2c and d). Although P13 CM-HCECs could not fully cover the wound within 12 h, it still had a faster rate than P3 BM-HCECs (Fig. 2c and d).

### Cultivated HCEC injection in the rabbit model

**Fig. 5:**
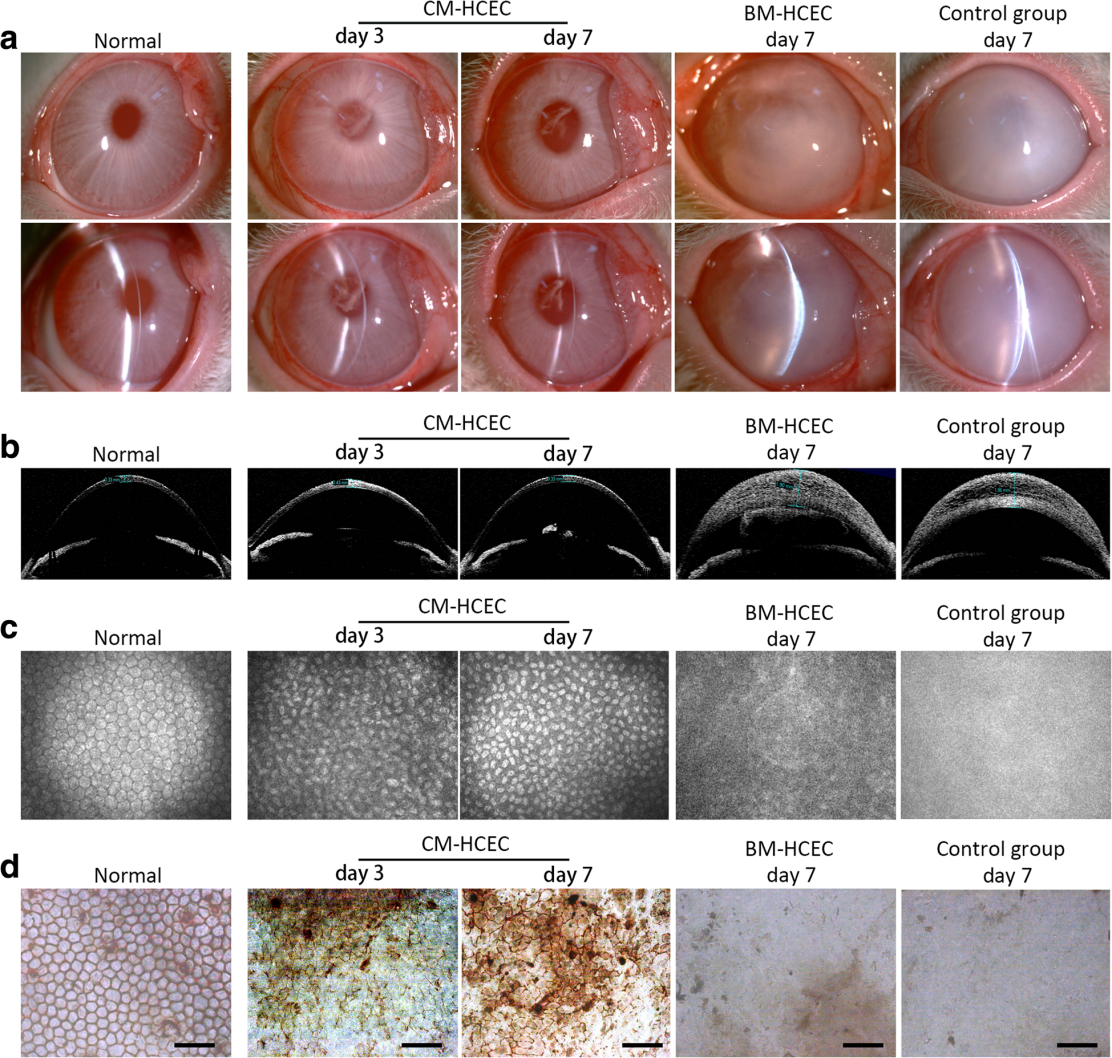
Injection and observation of HCECs in a rabbit corneal endothelial dysfunction model. **a** Slit-lamp photographs showed the clarity of the corneas in the P9 CM-HCEC group, the P3 BM-HCEC group, and the control group. **b** OCT showed differences in corneal thickness in the different groups. **c** Endothelium was detected by confocal microscope. **d** Alizarin Red S and trypan blue staining showed polygonal CECs in the P9 CM-HCEC group. No visible cells were found in the P3 BM-HCEC group and the control group. *Scale bars* = 100 μm. *BM* basal medium, *CM* conditioned medium, *HCEC* human corneal endothelial cell

**Fig. 6:**
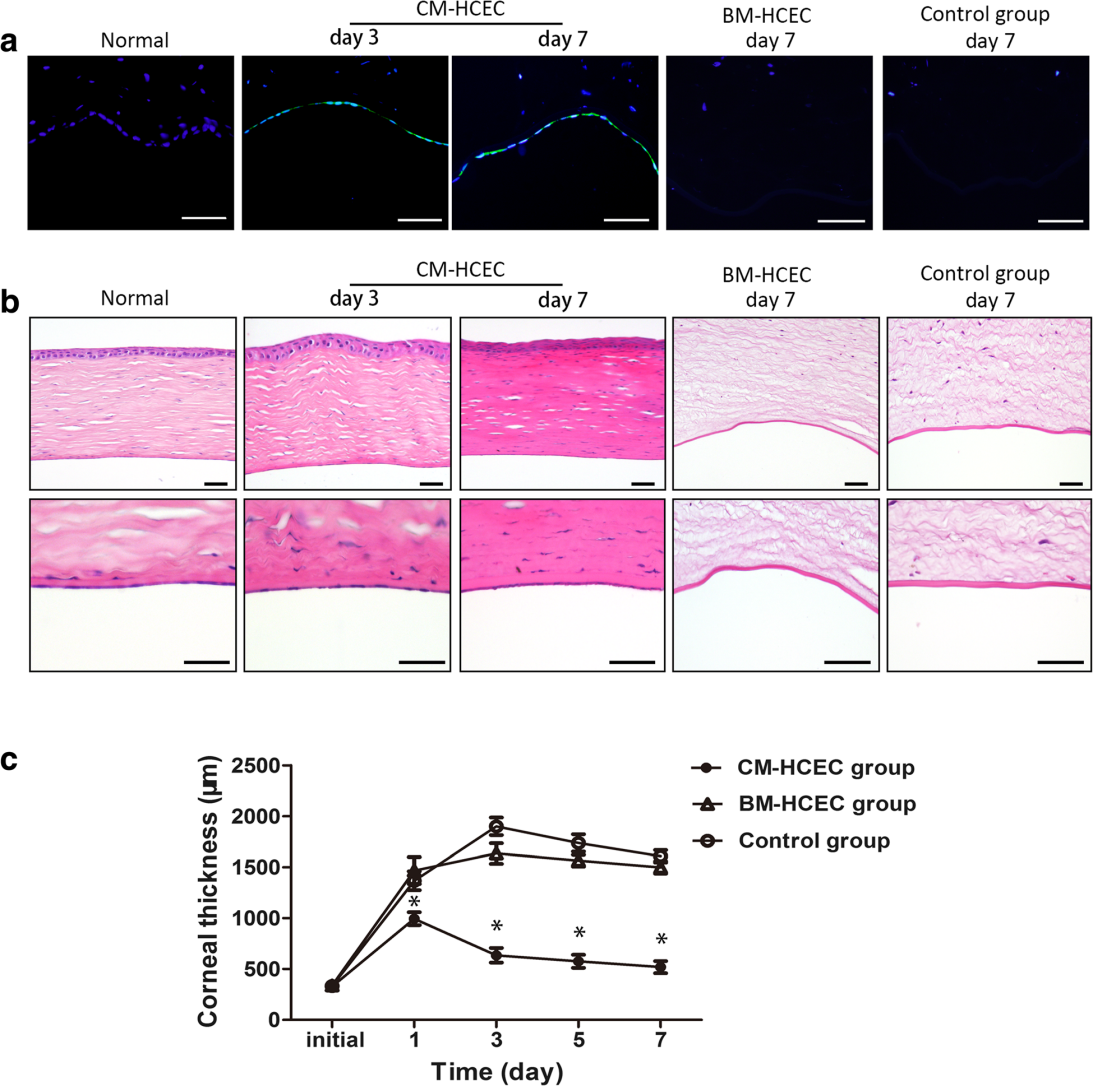
Histological examination and corneal thickness of rabbit corneas after the cell injection. **a** Detection of CFSE in different groups (*blue*: DAPI, *green*: CFSE). **b** H&E staining of corneas in the different groups. **c** Changes in the CCT was evaluated by OCT. The CCT in control eyes (*n* = 10) and in the eyes injected with BM-HCEC (*n* = 5) were significantly thicker than the CM-HCEC group (*n* = 20) at days 1, 3, 5, and 7. Results are means ± SEM. *p < 0.05. *Scale bars* = 100 μm. *BM* basal medium, *CM* conditioned medium, *HCEC* human corneal endothelial cell

To evaluate the repair ability of CM-HCECs in vivo, we use the rabbit corneal endothelial dysfunction model by removing endothelial cells from the DM and anterior chamber after which we performed cell injection (Additional file 1: Figure S1A and C). No residual host CECs were found in the collected aqueous (Additional file 1: Figure S1E and F). Preliminary results showed that CM-HCECs (before P13) were successful in recovering transparency of the endothelially damaged corneas within 7 days, although anterior chamber exudation could be seen (Fig. 5a). The corneal thickness rapidly decreased after injection of CM-HCECs (Fig. 5b). The changes in the central corneal thickness (CCT) were compared between different groups (Fig. 6c). Confocal microscope images and Alizarin Red S staining confirmed coverage of polygonal cells on the DM in the CM-HCEC group (Fig. 5c and d). The cell density of different groups were 2898 cells/mm^2^ (normal), 2008 cells/mm^2^ (P9 CM-HCECs, day 3), 2654 cells/mm^2^ (P9 CM-HCECs, day 7). In contrast, the control group in which the endothelium was only scraped induced severe corneal opacity and edema (Fig. 5a). P3 BM-HCECs could also not make the cornea transparent. Obvious graft rejection was also observed (Fig. 5a and b). The same result occurred in the rabbit model injected with P5 BM-HCECs (Additional file 2: Figure S2). There were no CECs detected in the BM-HCEC group and the control group by confocal microscopy and Alizarin Red S (Fig. 5c and d). Histological examination was performed after the euthanasia of the rabbits. The CFSE-labeled HCECs were detected by fluorescent microscope examination in the CM-HCEC group (Fig. 6a). H&E staining showed that the HCECs tightly adhered to the DM of the cornea in a monolayer (Fig. 6b), whereas the DMs in the BM-HCEC and control groups were bare and no cells were detected (Fig. 6a and b).

### Cultivated HCEC injection in the primate model

**Fig. 7:**
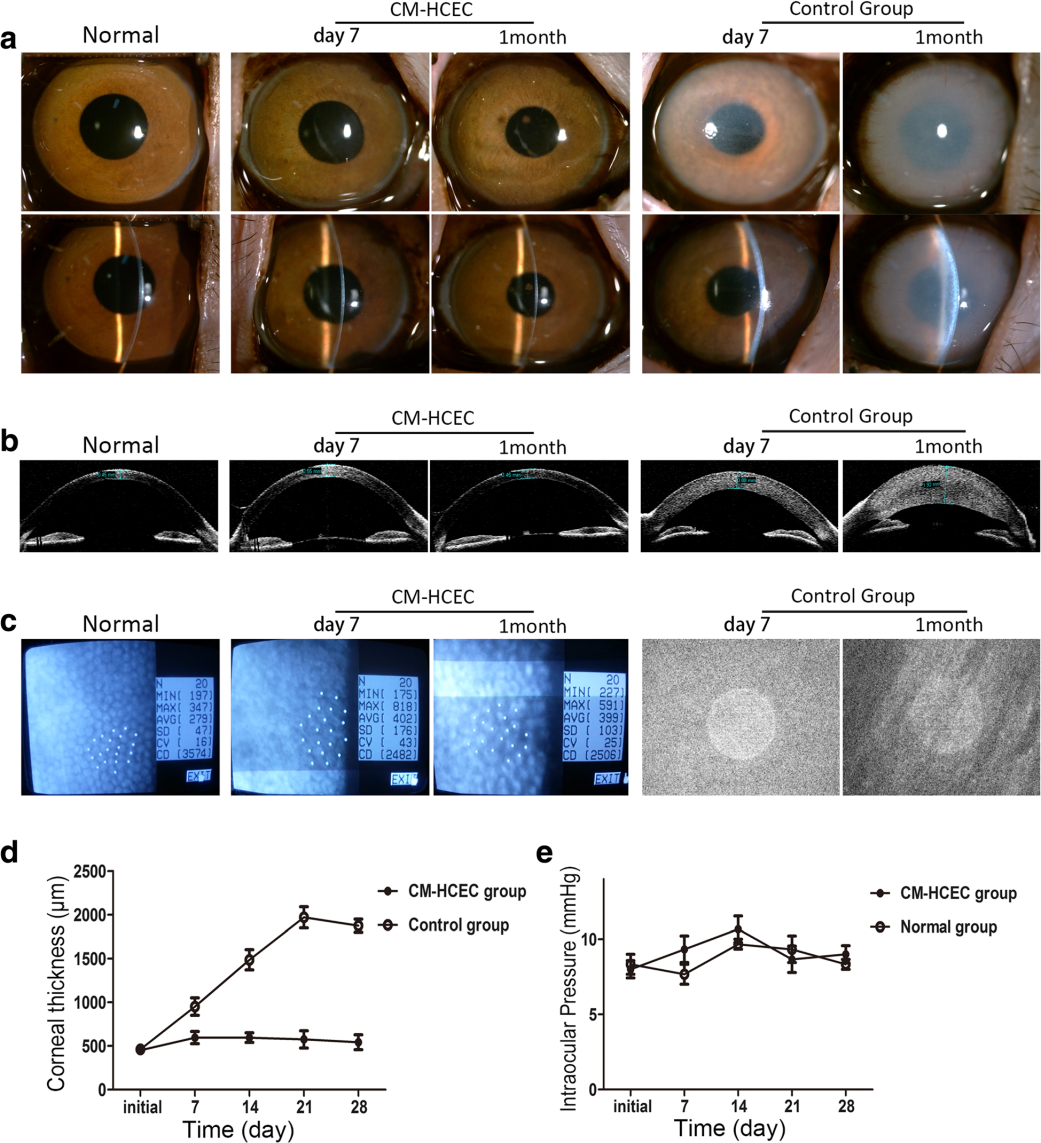
Preclinical research of cultured HCEC (P9 CM) injection in a monkey corneal endothelial dysfunction model. **a** Corneal transparency was examined by slit-lamp in the different groups. **b** OCT showed corneal thickness differences in the CM-HCEC group and the control group. **c** CECs were detected by noncontact specular microscopy. **d** Mean CCT was measured before and after the surgery. **e** Changes in IOP during the observation. Results are means ± SEM. *CM* conditioned medium, *HCEC* human corneal endothelial cell

For further research we used a monkey corneal endothelial dysfunction model to study the cell repair ability of CM-HCECs in vivo. Slit-lamp microscopy and OCT showed that the cells made the cornea transparent about 7 days after the injection. In contrast, the control group still had severe corneal opacity and stromal edema at 1 month after scraping the endothelium (Fig. 7a and b). Rejection reactions such as keratic precipitates (KP) and anterior chamber exudation were noticed days 10 to 14 after the injection, but an almost normal cornea and anterior chamber could be seen after the postoperative treatment (Additional file 3: Figure S3). One month after the surgery, the corneas of the CM-HCEC group became more transparent and thinner. On the contrary, the corneal opacity and stromal edema became more serious in the control group (Fig. 7a and b). CM-HCECs created a polygonal monolayer with a cell density of about 2500 cells/mm^2^. However, the corneal endothelium could not be clearly detected with specular microscopy or confocal microscopy in the control group because of the corneal opacity and edema (Fig. 7a–c). The CCT of the CM-HCEC group was much thinner than that of the control group after surgery, and almost the same as that in normal corneas (Fig. 7d). Intraocular pressures (IOP) were found to be no different between the CM-HCEC group and normal group (Fig. 7e).

**Fig. 8:**
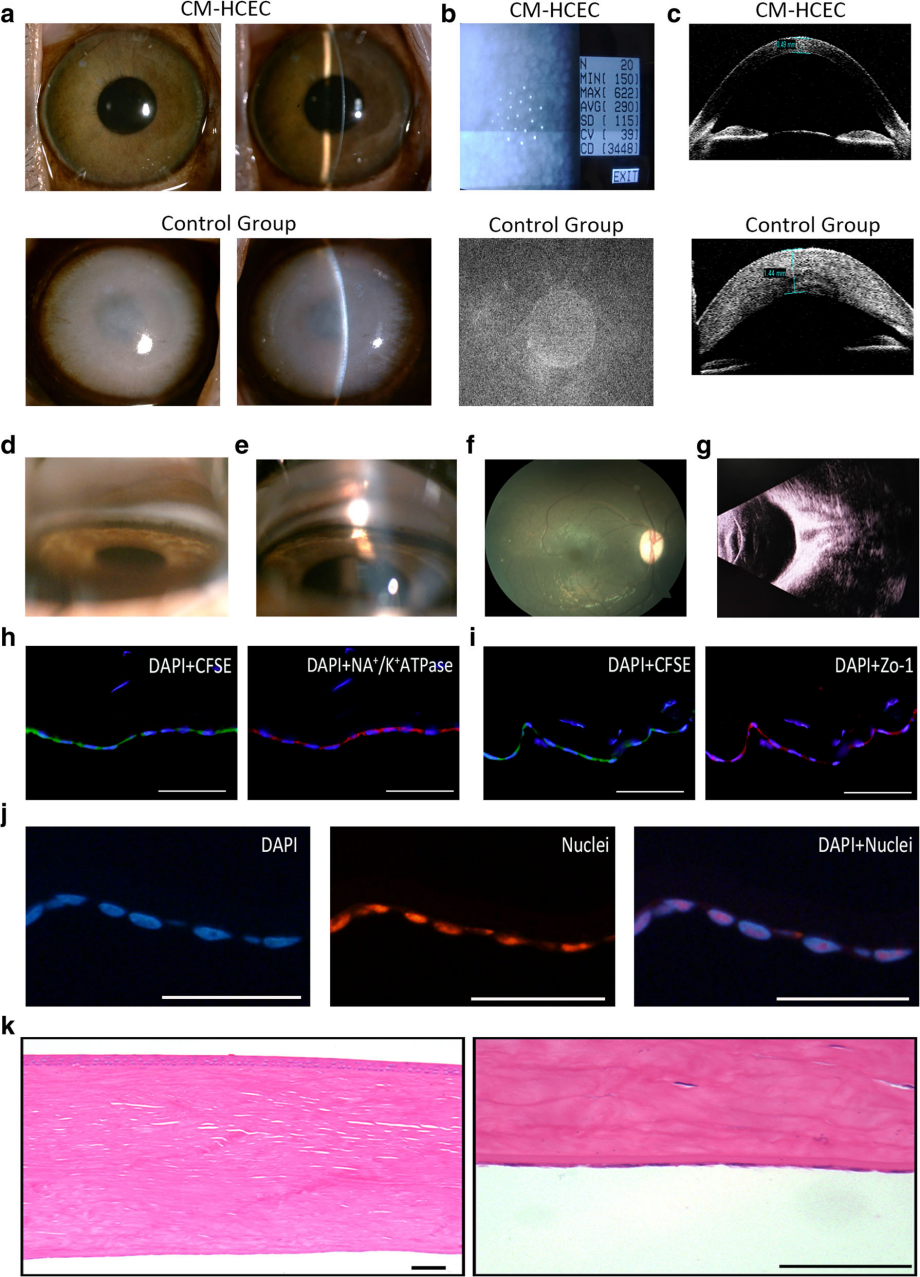
Long-term observation after CM-HCEC (P11) injection in the monkey model. **a** Corneal transparency was examined by slit-lamp in CM-HCEC group and control group. **b** CECs were detected by noncontact specular microscopy. **c** Corneal thickness differences in the CM-HCEC group and the control group by OCT. Angle images in the normal group (**d**) and the CM-HCEC group (**e**). **f** Fundus photography in the CM-HCEC group. **g** B-mode ultrasound in the CM-HCEC group. **h** Immunofluorescent staining of Na^+^/K^+^ ATPase (*blue*: DAPI, *green*: CFSE, *red*: Na^+^/K^+^ ATPase). **i** Immunofluorescent staining of Zo-1 (*blue*: DAPI, *green*: CFSE, *red*: Zo-1). **j** Immunofluorescent staining of nuclei (*blue*: DAPI, *red*: nuclei). **k** H&E staining of cornea in the CM-HCEC group. Images (**a**–**g**) were obtained 10 months after injection. Images (**h**–**k**) were obtained 2 months after injection. *Scale bars* = 100 μm. *CFSE* carboxyfluorescein succinmidyl ester, *CM* conditioned medium, *HCEC* human corneal endothelial cell, *Zo-1* zonula occudens-1

The monkey models were observed for 10 months to evaluate the long-term effects of the HCEC injection. The corneas of the CM-HCEC group were still as transparent as normal cornea, while corneal opacity and stromal edema were obvious in the control group (Fig. 8a and c). The CECs showed a mosaic pattern with a cell density of about 3400 cells/mm^2^ (Fig. 8b). A gonioscope, fundus photography, and B-mode ultrasound showed no pathological changes in the CM-HCEC eyes (Fig. 8d–g). Immunofluorescent staining showed CFSE-positive signals of HCECs and positive fluorescent staining of the nuclei 2 months after the cell injection, which demonstrated that the injected HCECs regenerated the corneal endothelium (Fig. 8h–j). Na^+^/K^+^ ATPase and Zo-1 were also expressed in HCECs, indicating the persistent pump and barrier function (Fig. 8h and i). H&E staining showed that the HCECs formed a monolayer on the DM (Fig. 8k). Immunohistochemical staining showed that no apparent human cells were found in other organs of the postoperative animals (Additional file 4: Figure S4). In addition, no obvious abnormalities were found in organs by H&E staining (Additional file 5: Figure S5).

## Discussion

As a major cause of corneal-related blindness, corneal endothelial dysfunction causes great damage to the visual acuity of a patient [6, 26]. Corneal transplantation is still the only effective treatment for this disease. In recent years, some new procedures such as Descemet’s stripping endothelial keratoplasty (DSEK), Descemet’s stripping automated endothelial keratoplasty (DSAEK), and Descemet’s membrane endothelial keratoplasty (DMEK) have been developed [6, 27–29]. Doctors and patients may have more choice and may even achieve better results. However, these methods face some obstacles such as their technical difficulty, continuing cell loss and detached lamellae after the operation, and graft rejections [30–32]. In addition, the worldwide shortage of donor corneas is a particularly large problem. Thus the concept of using one donor cornea for treating one patient is already obsolete because of the rapid development in regenerative medicine and tissue engineering [9]. Regardless of the treatments, the importance of sufficient quantities of functional HCECs is self-evident. However, it is difficult to establish the optimum method for isolation and cultivation of HCECs in vitro.

Thus, many researchers including us have explored using other cells, such as neural crest cells (NCCs) [11], embryonic stem cells (ESCs) [10, 33], adipose-derived stem cells (ADSCs) [34], bone marrow-derived endothelial progenitor cells [35], umbilical cord blood-derived mesenchymal stem cells (UCB-MSCs) [12], corneal stroma stem cells [36], and skin-derived precursors (SKPs) [37], as CEC-like cells. These CEC-like cells have similar shapes and some characteristics of native HCECs. However, the limitations of the proliferation ability, pump and barrier function, ethical issues, or lack of preclinical studies impedes the potential therapy in clinical applications of these introduced CECs-like cells [38, 39]. Therefore, the focus is still on how to improve proliferation and function of HCECs.

Recently, Nakahara et al. reported that HCEC expansion could be promoted by a conditioned medium obtained from human bone marrow-derived mesenchymal stem cells (BM-MSCs) [40]. An inhibitor to the transforming growth factor-beta receptor and a ROCK inhibitor also have beneficial effects on the expansion of HCECs [14, 41–43]. Kim and colleagues also compared the effects of different media on HCECs and attempted to find the optimum one [44]. However, we are still faced with difficulties with HCECs, such as the limited proliferative capacity and EMT with loss of function after a few passages [8, 45–47].

OASCs are derived from neural crest cells of the neuroectoderm [48, 49]. They possess a powerful proliferative capacity and multilineage differentiation potential, as do many other stem cells [18]. OASCs can be subcultured several times while maintaining the characteristics of stem cells. In addition, OASCs share the same embryonic origin with CECs [48]. We hypothesized that human OASCs could promote the expansion and function of HCECs while maintaining their phenotype. Besides, orbital adipose tissues are easily accessible and they provide huge numbers of stem cells [50]. We isolated HCECs and cultivated them with OASC-CM. The results of in vitro experiments showed that CM-HCECs have a polygonal shape similar to corneal endothelial cells in vivo, display an increased proliferative capacity, and highly express CEC-relative markers (N-cadherin, Na^+^/K^+^ ATPase, ZO-1, Col8a2, and SLC4A4) even after 10 passages. In order to evaluate the repair capacity of the cultured HCECs we conducted experimental cell injection into a rabbit corneal endothelial dysfunction model. The results showed that the cornea could obtain clarity within 7 days. However, the rabbit CECs have proliferative ability [51, 52]. Corneal transparency may be the result of the combined action between injected cells and residual host CECs from the far peripheral cornea even though almost all of the rabbit CECs were removed before the cell injection.

Literature reports that the monkey cornea is covered by migration of the remaining CECs when the corneal endothelium is injured, much like humans [5, 53, 54]. On the basis of the rabbit experiments we used a monkey model for further study aiming to eliminate the influence of proliferative CECs. The result of the primate experiment proved that the injection of CM-HCECs could well recover and maintain corneal transparency in corneal endothelial dysfunction models. Immunofluorescence and histological examination showed that CM-HCECs could form a monolayer of polygonal cells and regenerate functional corneal endothelium. After 10 months of postoperative observation, the corneas were still transparent and maintained the normal corneal thickness in the CM-HCEC group, while the corneas remained severely edemic in the control group. All the results of the present study indicated that HCECs cultivated with OASC-CM in vitro had good proliferative and therapeutic capacity in vivo.

Theoretically, if HCECs are isolated from one donor cornea and cultivated as P0 with OASC-CM, we can obtain about 7 × 10^5^ HCECs when they become confluent. When the cells are subcultured for 10 passages (passaged at a 1:2 ratio), the magnitude of HCECs would be 7 × 10^8^, and then 2500 eyes could be injected (each eye needs 2.7 × 10^5^ HCECs). These are encouraging prospects for clinical application.

The exact mechanisms of OASC-CM in promoting the proliferative and repair capacity of HCECs are not so clear. We hypothesize that the results are due to cytokines secreted by OASCs such as epidermal growth factor (EGF), basic fibroblasts growth factor (BFGF), and nerve growth factor (BGF). The HCECs then obtain some stem cell-like properties by using OASC-CM. This is one issue on which we should focus. Although corneal transparency recovered without adverse effects (secondary glaucoma or aberrant ectopic cell transplantation) in this study, episodes of immune rejection in some animals have been seen such as keratic precipitate (KP) and anterior chamber effusion after the cell injection; however, these are moderate and could be controlled by conventional therapy. The cause may be heterologous grafts, use of serum-containing medium, and nonadherent cells. This is another issue with which we should deal. Since we have a mature technological process for fabricating scaffold materials [55–58], we will explore cultivating HCECs with serum-free medium and seeding them in the scaffold materials to construct a lamellar or full thickness tissue-engineered corneal substitute which may have good prospects in future clinical applications.

## Conclusions

Use of the OASC-CM not only stimulates the proliferation of HCECs in vitro but also effectively promotes their repair capacity. We produced rabbit and monkey corneal endothelial dysfunction models and successfully treated them through cell injection. With this approach many more functional HCECs could be available for research and cell-based therapy for corneal endothelial dysfunction.

## Additional files

Additional file 1: Figure S1. Cultured HCEC injection in the corneal endothelial dysfunction model and detection of residual cells in collected aqueous of rabbits and monkeys. (A) Schema of the cell injection into anterior chamber. (1) Scrape of endothelium on DM. (2) Injection of cultured HCECs into the anterior chamber. (3) The animals were kept in the face-down position for 5 h to allow the HCECs to sink to the DM of the cornea. (C,D) The corneal endothelium was completely scraped from the DM (central and peripheral) of the rabbit model (C1 and 2) and monkey model (D1 and 2) with a modified irrigator needle (B). (C3 and D3) HCECs suspended in MEM were injected into the anterior chamber with an insulin needle. (E) Aqueous were stained by trypan blue and hematoxylin to detect residual cells. (F) Aqueous were cultured in 96-well plates. Scale bar = 50 μm. (TIF 5105 kb)

Additional file 2: Figure S2. Cultured HCEC (P5 BM and P5 CM) injection in a rabbit corneal endothelial dysfunction model. More eye drops (six times a day) and subconjuctival injection (every 2 days) of dexamethasone were given after surgery. The corneal transparency and thickness were observed and photographed by slit-lamp microscopy and OCT. (TIF 1784 kb)

Additional file 3: Figure S3. Cultured HCEC injection in a monkey corneal endothelial dysfunction model. Slit-lamp photographs showed the monkey corneal endothelial dysfunction model (left). Slit-lamp photographs showed the monkey corneal endothelial dysfunction model following injection of P11 CM-HCECs (right). Images were obtained at days 14 and 21 and months 2, 4, and 6 after surgery. (TIF 2490 kb)

Additional file 4: Figure S4. Immunohistochemical analysis of rabbit and monkey organs after surgery. (A) Immunohistochemical staining of human nuclei in rabbit organs. (B) Immunohistochemical staining of human nuclei in monkey organs. (TIF 7183 kb)

Additional file 5: Figure S5. H&E staining of monkey organs after the HCEC injection. Scale bar = 100 μm. (TIF 6389 kb)

## Abbreviations

ADSC: Adipose-derived stem cell
APC: Allophycocyanin
BM: Basal culture medium
BM-MSC: Bone marrow-derived mesenchymal stem cell
CCK-8: Cell counting kit-8
CE: Corneal endothelium
CEC: Corneal endothelial cell
CFSE: Carboxyfluorescein diacetate succinimdyl ester
CM: Conditioned medium
DM: Descemet’s membranes
DMEK: Descemet’s membrane endothelial keratoplasty
DMEM-LG: Dulbecco’s modified Eagle’s medium low glucose
DSAEK: Descemet's stripping automated endothelial keratoplasty
DSEK: Descemet’s stripping endothelial keratoplasty
EMT: Endothelial-to-mesenchymal transition
ESC: Embryonic stem cell
FBS: Fetal bovine serum
FITC: Fluorescein isothiocyanate
H&E: Hematoxylin and eosin
HCEC: Human corneal endothelial cell
hEGF: Human epidermal growth factor
KP: Keratic precipitates
NCC: Neural crest cell
OASC: Orbital adipose-derived stem cell
OCT: Optical coherent tomography
PBS: Phosphate-buffered saline
PE: Phycoerythrin
qRT-PCR: Quantitative real-time reverse transcription polymerase chain reaction
RT: Room temperature
SKP: Skin-derived precursor
UCB-MSC: Umbilical cord blood-derived mesenchymal stem cell
ZO-1: Zonula occludens-1
