# Supplementary figures and images for: Promoting the expansion and function of human corneal endothelial cells with an orbital adipose-derived stem cell-conditioned medium
Source: Stem Cell Res Ther. 2017 Dec 20;8:287. doi: 10.1186/s13287-017-0737-5 (PMC5738836; doi:10.1186/s13287-017-0737-5)

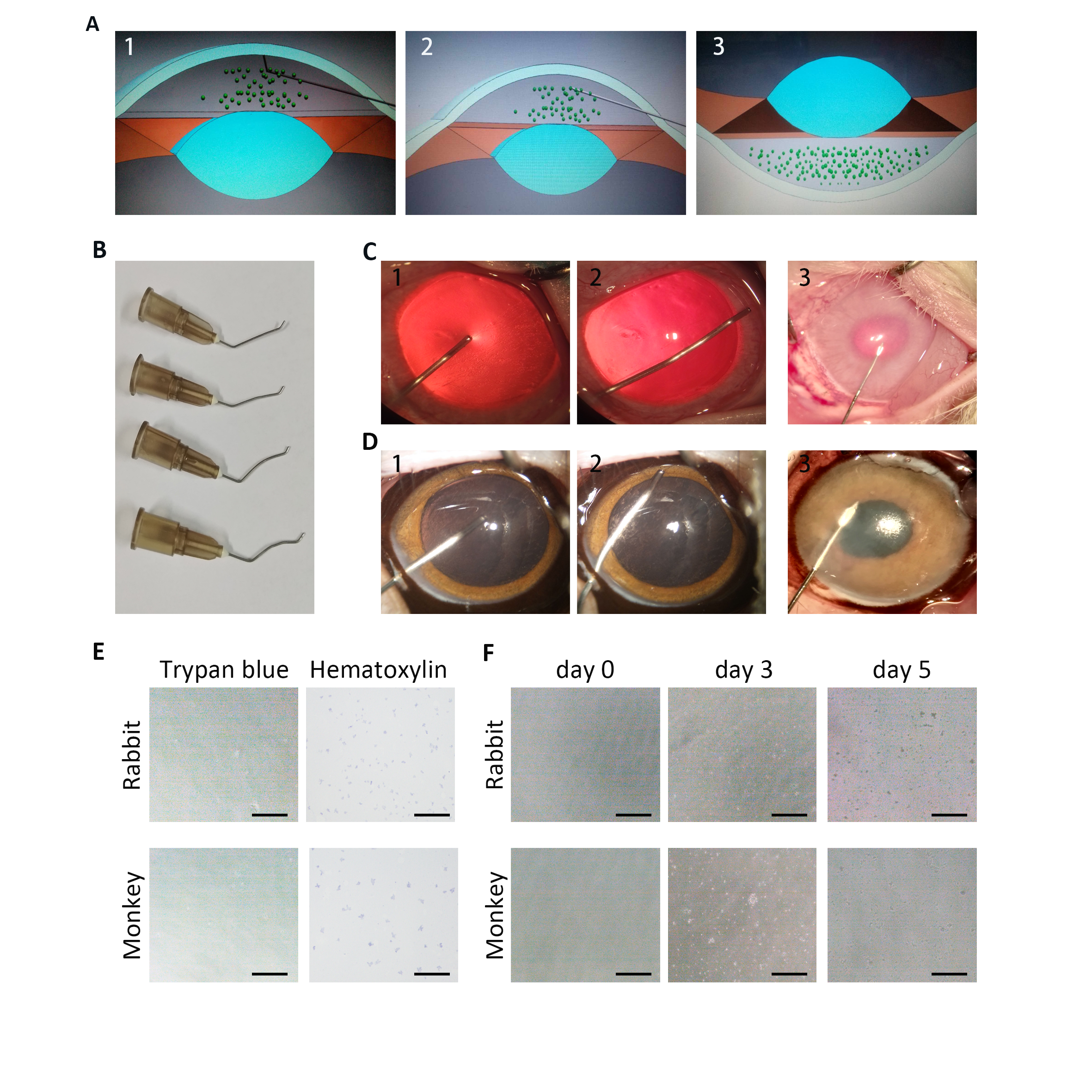

Supplement: Supplementary file 1 — Cultured HCEC injection in the corneal endothelial dysfunction model and detection of residual cells in collected aqueous of rabbits and monkeys. (A) Schema of the cell injection into anterior chamber. (1) Scrape of endothelium on DM. (2) Injection of cultured HCECs into the anterior chamber. (3) The animals were kept in the face-down position for 5 h to allow the HCECs to sink to the DM of the cornea. (C,D) The corneal endothelium was completely scraped from the DM (central and peripheral) of the rabbit model (C1 and 2) and monkey model (D1 and 2) with a modified irrigator needle (B). (C3 and D3) HCECs suspended in MEM were injected into the anterior chamber with an insulin needle. (E) Aqueous were stained by trypan blue and hematoxylin to detect residual cells. (F) Aqueous were cultured in 96-well plates. Scale bar = 50 μm. (TIF 5105 kb) [file 13287_2017_737_MOESM1_ESM.tif]

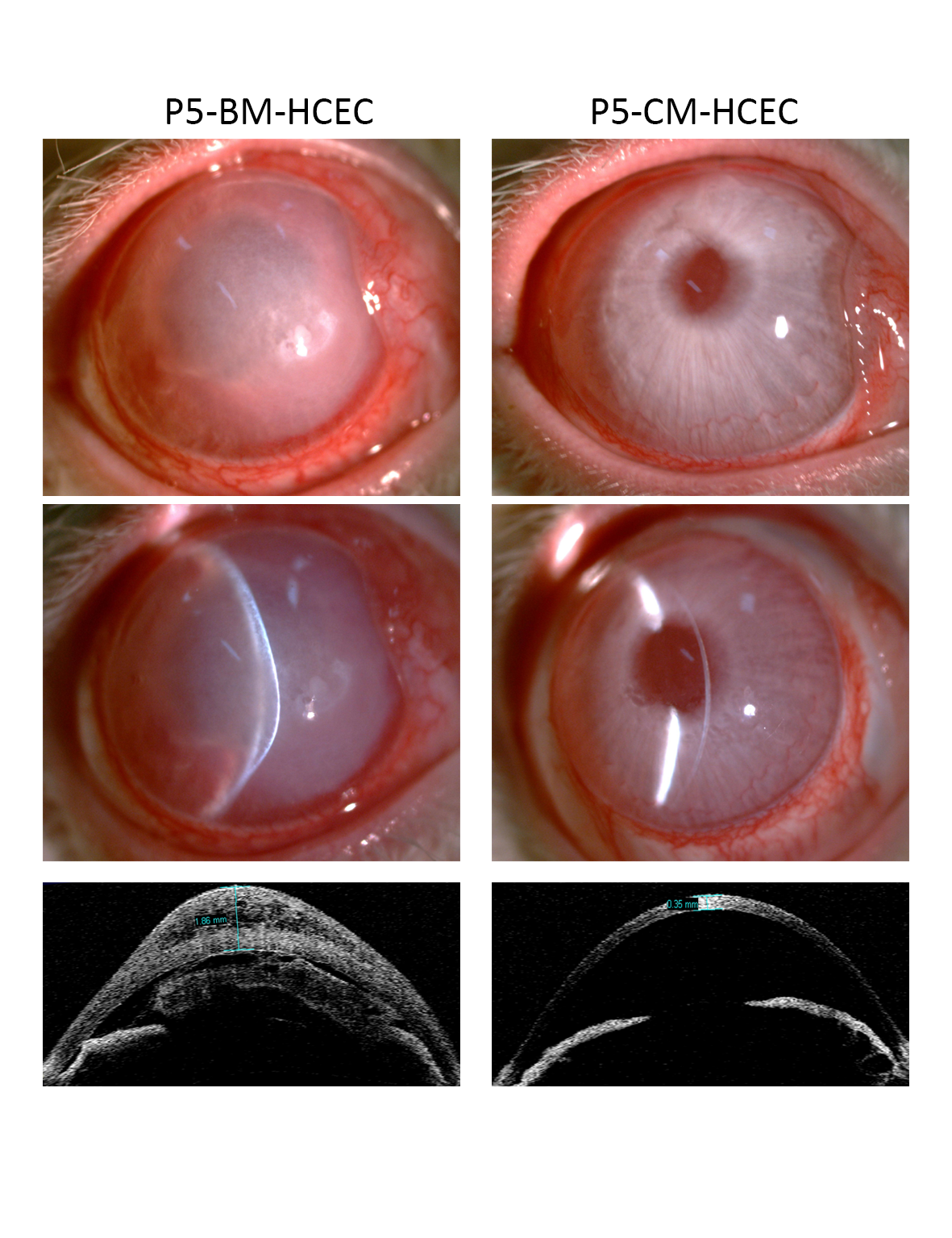

Supplement: Supplementary file 2 — Cultured HCEC (P5 BM and P5 CM) injection in a rabbit corneal endothelial dysfunction model. More eye drops (six times a day) and subconjuctival injection (every 2 days) of dexamethasone were given after surgery. The corneal transparency and thickness were observed and photographed by slit-lamp microscopy and OCT. (TIF 1784 kb) [file 13287_2017_737_MOESM2_ESM.tif]

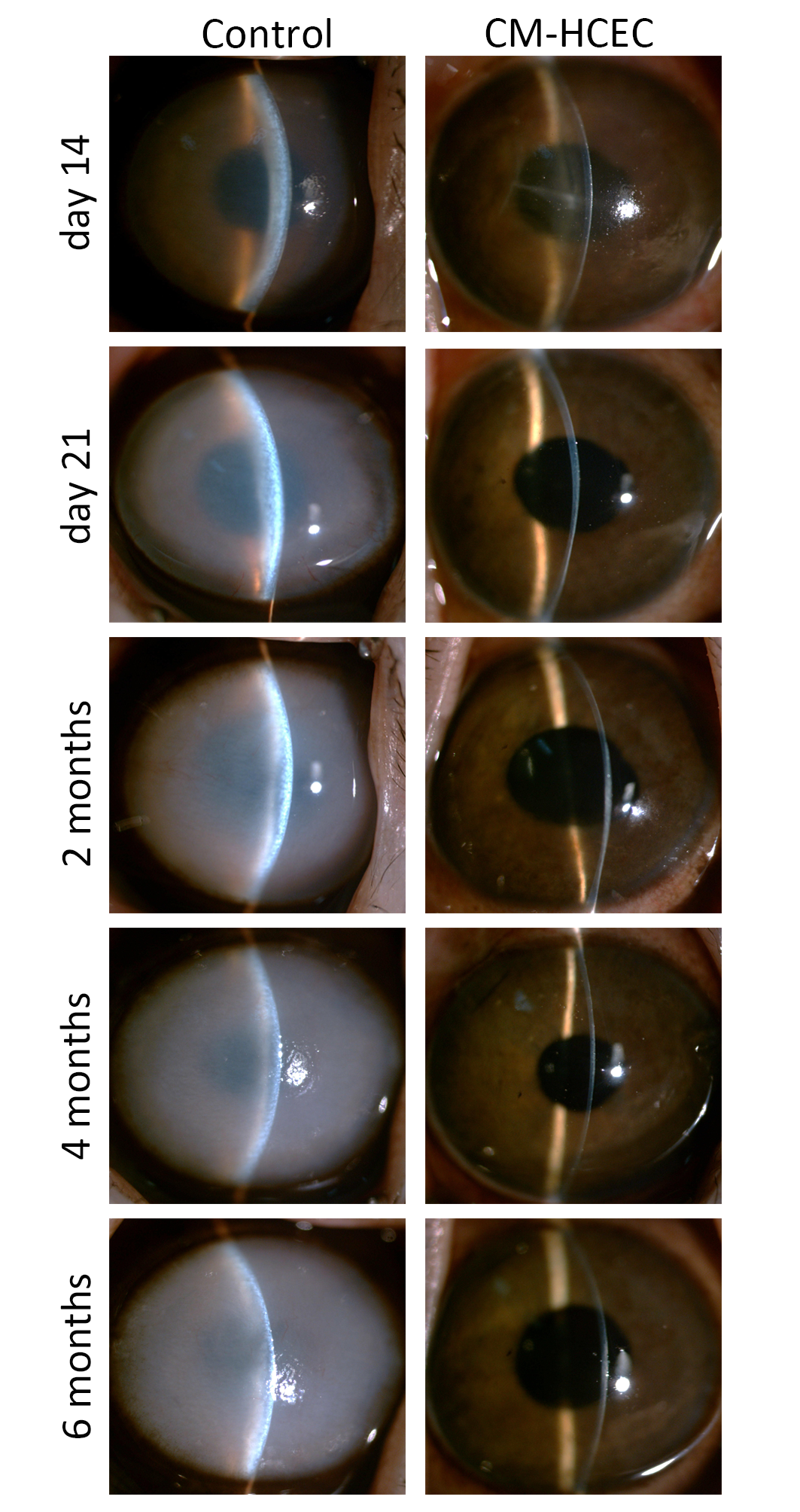

Supplement: Supplementary file 3 — Cultured HCEC injection in a monkey corneal endothelial dysfunction model. Slit-lamp photographs showed the monkey corneal endothelial dysfunction model (left). Slit-lamp photographs showed the monkey corneal endothelial dysfunction model following injection of P11 CM-HCECs (right). Images were obtained at days 14 and 21 and months 2, 4, and 6 after surgery. (TIF 2490 kb) [file 13287_2017_737_MOESM3_ESM.tif]

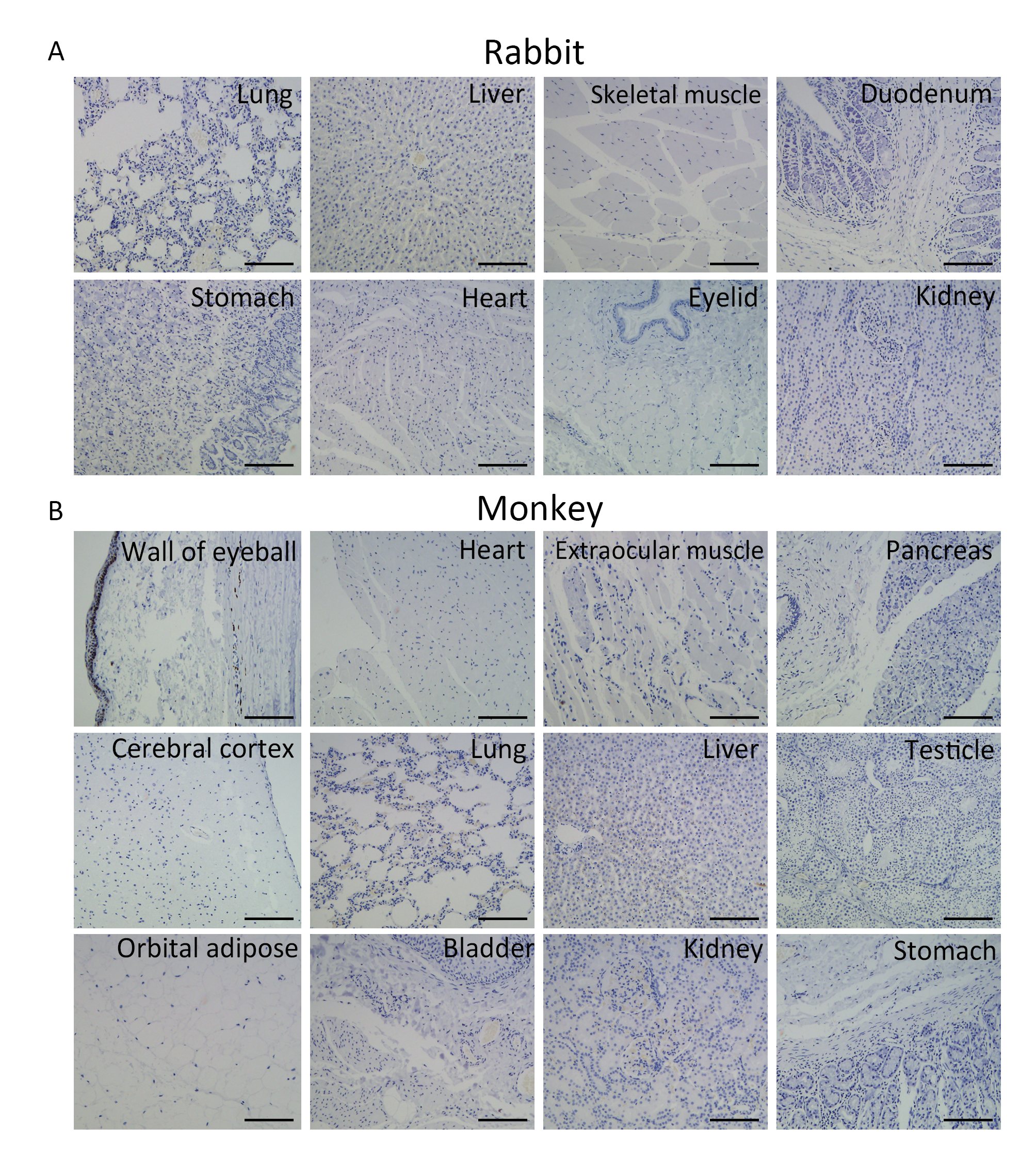

Supplement: Supplementary file 4 — Immunohistochemical analysis of rabbit and monkey organs after surgery. (A) Immunohistochemical staining of human nuclei in rabbit organs. (B) Immunohistochemical staining of human nuclei in monkey organs. (TIF 7183 kb) [file 13287_2017_737_MOESM4_ESM.tif]

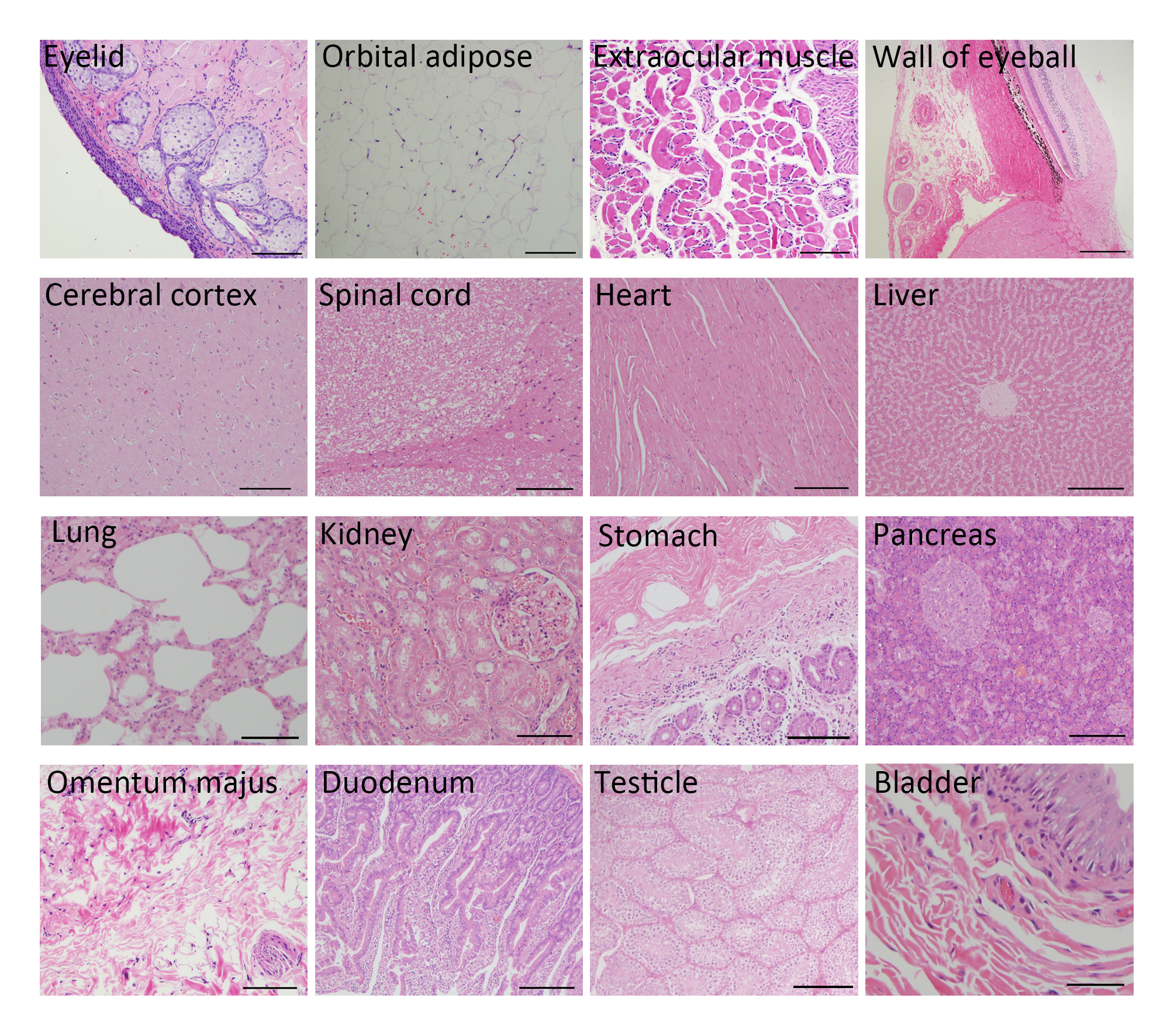

Supplement: Supplementary file 5 — H&E staining of monkey organs after the HCEC injection. Scale bar = 100 μm. (TIF 6389 kb) [file 13287_2017_737_MOESM5_ESM.tif]
